# Fatty acid transport protein-5 (FATP5) deficiency enhances hepatocellular carcinoma progression and metastasis by reprogramming cellular energy metabolism and regulating the AMPK-mTOR signaling pathway

**DOI:** 10.1038/s41389-021-00364-5

**Published:** 2021-11-12

**Authors:** Ming-Da Wang, Nan-Ya Wang, Hui-Lu Zhang, Li-Yang Sun, Qiu-Ran Xu, Lei Liang, Chao Li, Dong-Sheng Huang, Hong Zhu, Tian Yang

**Affiliations:** 1 Department of Hepatobiliary Pancreatic and Minimal Invasive Surgery, Zhejiang Provincial People’s Hospital (People’s Hospital of Hangzhou Medical College), Hangzhou, Zhejiang China; 2 Department of Hepatobiliary Surgery, Eastern Hepatobiliary Surgery Hospital, Second Military Medical University (Navy Medical University), Shanghai, China; 3 The Cancer Center, the First Hospital of Jilin University, Changchun, Jilin China; 4 Department of Digestive Diseases, Huashan Hospital, Fudan University, Shanghai, China; 5 School of Clinical Medicine, Hangzhou Medical College, Hangzhou, China; 6 Department of Medical Oncology, the First Affiliated Hospital of Soochow University, Suzhou, China

**Keywords:** Cancer metabolism, Liver cancer

## Abstract

Aberrant lipid metabolism is an essential feature of hepatocellular carcinoma (HCC). Fatty acid transport protein-5 (FATP5) is highly expressed in the liver and is involved in the fatty acid transport pathway. However, the potential role of FATP5 in the pathogenesis of HCC remains largely unknown. Herein, we showed that FATP5 was downregulated in HCC tissues and even much lower in vascular tumor thrombi. Low expression of FATP5 was correlated with multiple aggressive and invasive clinicopathological characteristics and contributed to tumor metastasis and a poor prognosis in HCC patients. FATP5 inhibited the epithelial–mesenchymal transition (EMT) process and suppressed HCC cell migration and invasion, while silencing FATP5 had the opposite effects. Mechanistically, knockdown of FATP5 promoted cellular glycolytic flux and ATP production, thus suppressing AMP-activated protein kinase (AMPK) and activating its downstream signaling mammalian target of rapamycin (mTOR) to support HCC progression and metastasis. Activation of AMPK using metformin reversed the EMT program and impaired the metastatic capacity of FATP5-depleted HCC cells. Collectively, FATP5 served as a novel suppressor of HCC progression and metastasis partly by regulating the AMPK/mTOR pathway in HCC, and targeting the FATP5-AMPK axis may be a promising therapeutic strategy for personalized HCC treatment.

## Introduction

Hepatocellular carcinoma (HCC) ranks as the fifth most common cancer globally and results in significant health-related problems, making it the third leading cause of cancer-related deaths [1]. Although early HCC can be cured by liver resection in selected candidates, most patients are still diagnosed at advanced stages for which potentially curative therapies are no longer considered [2]. Even for patients who have undergone curative resection for resectable or localized HCCs, the long-term outcomes remain dismal primarily because of the high incidences of tumor metastasis, with up to 70% of cases developing recurrence within 5 years after surgery [3]. Although multiple molecular targets and signaling pathways have been investigated in HCC, the definitive mechanisms underlying HCC development and progression remain elusive; however, effective antitumor agents have not been developed. Therefore, despite an increased understanding of the molecular pathogenesis and heterogeneity of HCC, a high unmet demand exists to identify novel therapeutic targets and pharmacological treatment options for HCC.

Metabolic reprogramming is an emerging potential hallmark of cancer [4]. In particular, alterations in fatty acid (FA) metabolism have been increasingly recognized in multiple malignancies, including HCC [5]. Concrete evidence has indicated that aberrant lipid metabolism is strongly related to HCC pathogenesis and contributes to various aspects of HCC development [6, 7]. Exacerbated de novo lipogenesis is believed to be the dominant metabolic phenotype involving HCC initiation and progression [8]. Additionally, fatty acid transport is also a general metabolic pathway and plays pivotal roles in hepatocarcinogenesis [9]. Fatty acid transport proteins (FATPs) are a class of intracellular proteins comprising an FA uptake and transport system with high affinity for long-chain FAs [10]. Among them, FATP5 is exclusively expressed in the liver and is involved in cellular FA transport and bile metabolism [11]. The results from Doege et al., using a FATP5-knockout mouse model, revealed that silencing FATP5 in vivo significantly improved whole-body insulin sensitivity and reversed fat-diet-induced obesity and even fatty liver disease [12]. Additionally, these mice failed to gain weight when fed high-fat diets because of reduced uptake of long-chain FAs and increased anabolic process of FAs in hepatocytes, further confirming the critical role of FATP5 in the maintenance of both glucose and lipid metabolism.

In addition to its major role in lipid metabolism, FATP5 is also involved in tumorigenesis. Previous studies have reported conflicting results regarding the expression levels of FATP5 in lung cancer [13], esophageal squamous cell carcinoma [14], and HCC [15], suggesting the multifunctional roles of FATP5 in different tumor types. A recent study demonstrated that aberrant DNA hypermethylation led to the downregulation of FATP5, which caused the accumulation of polyunsaturated lipids and subsequent production of reactive oxygen species in HCC cells. Furthermore, blocking the downstream pathway of FATP5 significantly rendered FATP5-deficient HCC cells more sensitive to sorafenib treatment [15]. These results primarily revealed the anticancer effect of FATP5 on HCC cells; however, the specific role and possible mechanisms by which FATP5 promotes HCC progression and metastasis remain largely unknown.

In the present study, we aimed to evaluate the impact of FATP5 on HCC progression and metastasis and explore the underlying mechanisms. Furthermore, potential therapeutic targets for patients with aberrant FATP5 expression were also investigated. We first observed that FATP5 was expressed at low levels in both HCC and tumor thrombus tissues, and decreased expression of FATP5 was positively associated with more invasive clinicopathological features and a worse prognosis in HCC patients, suggesting an anti-oncogenic role of FATP5 as a tumor suppressor in HCC progression. Furthermore, silencing FATP5 significantly promoted cell migration and invasion, as well as the epithelial-to-mesenchymal transition (EMT) process, in HCC cells and vice versa. Our findings also highlighted a novel FATP5-mediated AMP-activated protein kinase (AMPK) and mammalian target of rapamycin (mTOR) signaling pathway in HCC cells that regulates the invasive and EMT phenotype by reprogramming intracellular glycolysis and energy metabolism, thus providing potential therapeutic targets to develop treatment strategies for FATP5-dependent HCC.

## Results

### Downregulated expression of FATP5 is frequently detected in HCC tissues

**Fig. 1:**
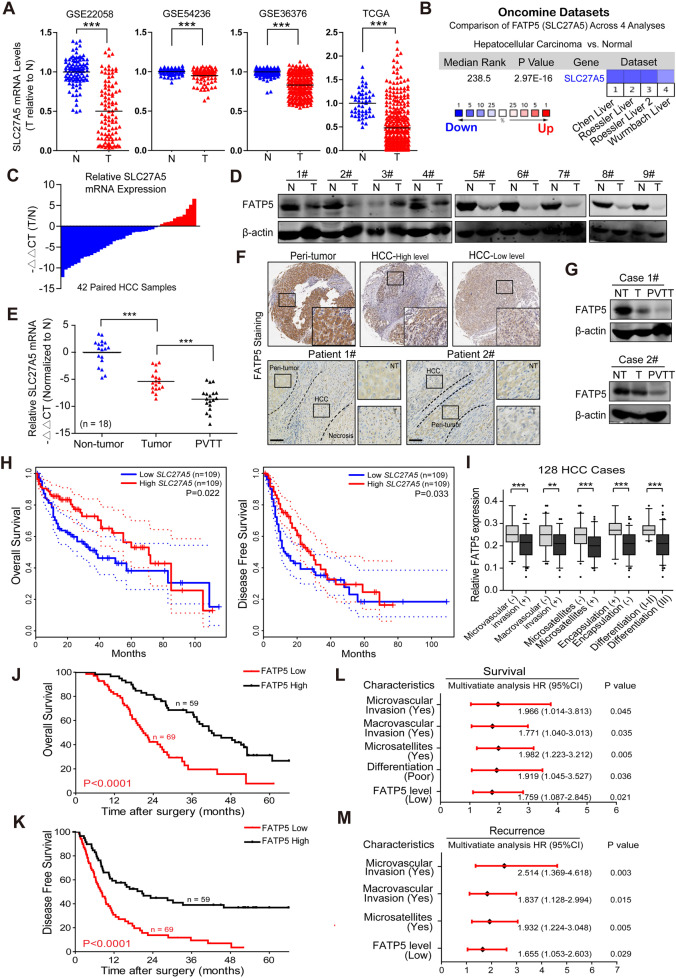
Downregulated expression of FATP5 is frequently detected in HCC tissues and correlates with aggressive clinicopathological features and predicts poor oncologic prognosis in HCC patients. **A** Relative mRNA levels of FATP5 (*SLC27A5*) in human HCC and normal liver tissues was analyzed using 4 HCC datasets (GSE22058, GSE54236, GSE36376, and TCGA Liver Hepatocellular Carcinoma dataset) and were shown by Box plots. Values represented as mean ± SD, ^***^P < 0.001, two-tailed *t*-test. **B** Other 4 independent studies published in ONCOMINE database were utilized to compare the mRNA expression of *SLC27A5* in HCC specimens and non-tumor tissues. **C** Relative mRNA levels of *SLC27A5* in 42 paired HCC and adjacent non-tumor tissues were evaluated by qRT-PCR and presented as −ΔΔCt values. **D** Western blotting was performed to detect FATP5 protein expression in HCC tissues (T) and their matched nontumoral tissues (N), and representative images were shown. β-actin was used as a loading control. **E** Relative mRNA expression of *SLC27A5* in 18 pairs of peri-tumoral, tumor and portal vein tumor thrombus (PVTT) tissues were assessed by qRT-PCR. All data were normalized to nontumoral group and presented as −ΔΔCt values. ^***^P < 0.001, two-tailed *t*-test. For all assays in (**C**) and (**E**), the GAPDH expression was used as an endogenous control to normalize the mRNA levels. **F** Representative immunohistochemistry (IHC) staining of FATP5 in the TMA specimens (normal liver, high- and low-FATP5-expressing HCCs; upper panel) and matched peri-tumoral and tumor tissues from 2 independent HCC patients after surgery (down panel). Scale bar: 50 μm. **G** Representative western blotting showed the protein level of FATP5 in matched peri-tumoral, tumor and PVTT tissues. β-actin was used as a loading control. **H** Kaplan-Meier curves for overall survival (OS; left) and disease-free survival (DFS; right) rates of HCC patients in TCGA cohort. According to the mRNA levels of *SLC27A5*, HCC patients were divided into high- (top 30%) and low- (bottom 30%) *SLC27A5*-expressing groups, respectively. **I** Relative expression levels of FATP5 in 128 human HCC samples with or without micro-/macrovascular invasion, microsatellites, incomplete encapsulation and poor tumor differentiation. Values represented as mean ± SD, ^**^P < 0.01, and ^***^P < 0.001, two-tailed *t*-test. **J**, **K** The OS and DFS rates in HCC patients with low- and high-FATP5 expression were analyzed by Kaplan-Meier’s method (*P* < 0.05 by log-rank test). **L**, **M** Multivariate analyses of the hazard ratios (HRs) for patient survival and tumor recurrence after curative resection. All enrolled variables were selected using univariate analyses and the HRs were presented as the means (95% confidence interval).

To assess the functional role and clinical significance of FATP5 (also termed *SLC27A5*) in HCC progression, we first analyzed FATP5 expression in HCC patients using public datasets from TCGA, GEO, and ONCOMINE. A lower FATP5 mRNA expression was observed in HCC specimens than in matched nontumoral tissues (Fig. 1A, B and Supplementary Fig. 1). To validate these findings, we examined FATP5 expression in paired HCC and noncancerous samples by qRT-PCR and western blot. Both the mRNA and protein expression levels of FATP5 were significantly lower in most HCC samples than in their corresponding nontumoral tissues (Fig. 1C, D). Previous studies have indicated a strong correlation between portal vein invasion and a dismal prognosis of HCC, and patients with portal vein tumor thrombus (PVTT) were more likely to develop intra/extrahepatic metastases even after curative treatments and, therefore, had a more aggressive disease course and worse clinical outcomes [16]. We next compared the FATP5 expression level in PVTT, HCC, and adjacent non-HCC tissues and found that the FATP5 mRNA level was more decreased in PVTT samples than in either the corresponding HCC or the peri-tumoral tissues (Fig. 1E). Similar results were also obtained by immunohistochemistry (IHC) staining and western blotting, with representative images shown in Fig. 1F, G. Taken together, FATP5 was frequently downregulated in human HCCs, particularly in tumor thrombus tissues, indicating a potential role of FATP5 in inhibiting HCC progression and metastasis.

### Decreased FATP5 expression correlates with aggressive clinicopathological features and predicts poor oncologic prognosis in HCC patients

We next explored the relationship between FATP5 expression and patient survival using consecutive data of the HCC population from the TCGA database. HCC patients with low FATP5 expression had poorer overall survival (OS) and disease-free survival (DFS) than those with high expression levels (Fig. 1H). Additionally, significantly lower FATP5 expression was correlated with advanced tumor stages, the presence of satellites and vascular invasions in different HCC datasets (Supplementary Fig. 2A–C). To validate these results and further investigate the association between FATP5 and the clinicopathological features of HCC patients, we first performed IHC staining to detect FATP5 expression in HCC tissue microarrays (TMAs) containing 128 paired tissues. Depending on the calculated staining intensity of each sample in the TMA, all 128 patients were divided into high (*n* = 59) and low (*n* = 69) FATP5 groups, and details of their clinicopathological features are listed in Supplementary Tables 1 and 2. No remarkable relationship was found between FATP5 expression and the tumor number, maximum tumor diameter, or serum AFP level (Supplementary Fig. 2D), but the downregulation of FATP5 in HCC was significantly associated with several aggressive clinical phenotypes, such as micro- and macrovascular invasion, satellites, incomplete tumor encapsulation and poor differentiation (Fig. 1I), supporting the critical role of FATP5 in HCC invasion and metastasis. Furthermore, patients in the FATP5-low group exhibited shorter tumor-free survival times and worse survival rates than those in the FATP5-high group (Fig. 1J, K). Additionally, multivariate analysis further noted that low expression levels of FATP5, together with microsatellites and micro- and macrovascular invasion, were independent risk factors for both OS and DFS in HCC patients (Fig. 1L, M and Supplementary Table 3). Taken together, these results suggested that the FATP5 expression level of FATP5 serves as an effective prognostic predictor for recurrence and survival of HCC patients, and supports the critical role of FATP5 in HCC invasion and metastasis.

### FATP5 suppresses the invasion and metastasis of HCC cells both in vitro and in vivo

**Fig. 2:**
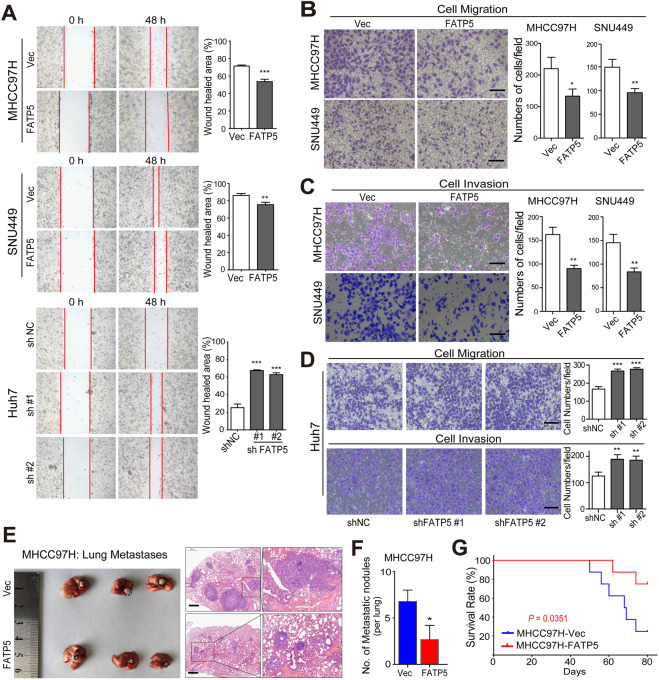
FATP5 suppresses the invasion and metastasis of HCC cells both in vitro and in vivo. **A** Scratch wound-healing assays were performed to evaluate the cell motility in both FATP5-overexpressing and FATP5-silencing HCC cells. After scratching the surface of cell layers, representative images were captured using microscope at 48 h (magnification, 100×) and the wound healed area were calculated according to the remained scratch size at 48 h compared to that area at the beginning. **B**–**D** Cell migration and invasion assays were performed to evaluate the migratory and invasive properties. After incubation for 48 h, the migrated and invaded cells were fixed with crystal violet and the cell numbers were microscopically counted. Representative images were shown and results were presented as the average number of cells from 5 independent microscopic fields. Scale bar: 50 μm. All values in (**A**–**D**) represented as mean ± SD, ^*^P < 0.05, ^**^P < 0.01, and ^***^P < 0.001. **E**, **F** A nude mouse lung metastasis model was established based on tail vein injection of 1 × 10^6^ MHCC97H-Vec and -FATP5 cells. Representative images (left panel) and H&E stained sections (right panel) showing visible and microscopic metastatic nodules in lung of mice in the MHCC97H-Vec and FATP5-expressing groups (**E**). The numbers of lung metastatic foci with diameter ≥3 mm in mice were counted and presented as mean ± SD, ^*^P < 0.05 compared with the MHCC97H-Vec group (**F**). Scale bar: 500 μm. **G** Comparisons of OS curves in mice injected with either MHCC97H-Vec or MHCC97H-FATP5 cells were analyzed by Kaplan-Meier’s method (*P* < 0.05 by log-rank test).

Given the significant relevance between FATP5 expression and the invasive clinical features of HCC patients, we speculated that FATP5 plays an antitumor role in HCC progression and metastasis, and the impact of FATP5 on the migration and invasion of HCC cells was evaluated. According to the endogenous FATP5 levels in multiple hepatoma cell lines (Supplementary Fig. 3A), we established stable FATP5-overexpressing and FATP5-silenced HCC cell lines using FATP5-encoding plasmids and lentivirus-based short hairpin (sh) RNAs, respectively. The efficiencies of overexpression and knockdown were confirmed at both the mRNA and protein levels (Supplementary Fig. 3B, C). Compared with control cells (referred to as Vec), enforced expression of FATP5 significantly suppressed cell motility and invasive capacity, as shown by wound-healing, migration, and Matrigel invasion assays (Fig. 2A–C), whereas FATP5 interference abolished these effects, with evidently higher levels of cell migration and invasion in shFATP5-transfected Huh7 cells (Fig. 2A, D). We next validated these findings in vivo by establishing a nude mouse lung metastasis model. Consistently, FATP5 overexpression significantly suppressed xenograft tumor formation and growth, as indicated by fewer and smaller micrometastatic lesions in lungs from mice injected with MHCC97H-FATP5 cells than in those injected with MHCC97H-Vec cells (Figs. 2E, F). Additionally, the mice inoculated with MHCC97H-FATP5 cells displayed a much higher survival rate (75% vs. 25%; *P* = 0.0351, with 80 days as the cutoff) (Fig. 2G). Collectively, these gain- and loss-of-function studies clearly revealed the critical role of FATP5 in regulating the invasiveness and highly metastatic features of HCC cells both in vitro and in vivo.

### FATP5 inhibits EMT by regulating AMPK-mTOR-S6K signaling

**Fig. 3:**
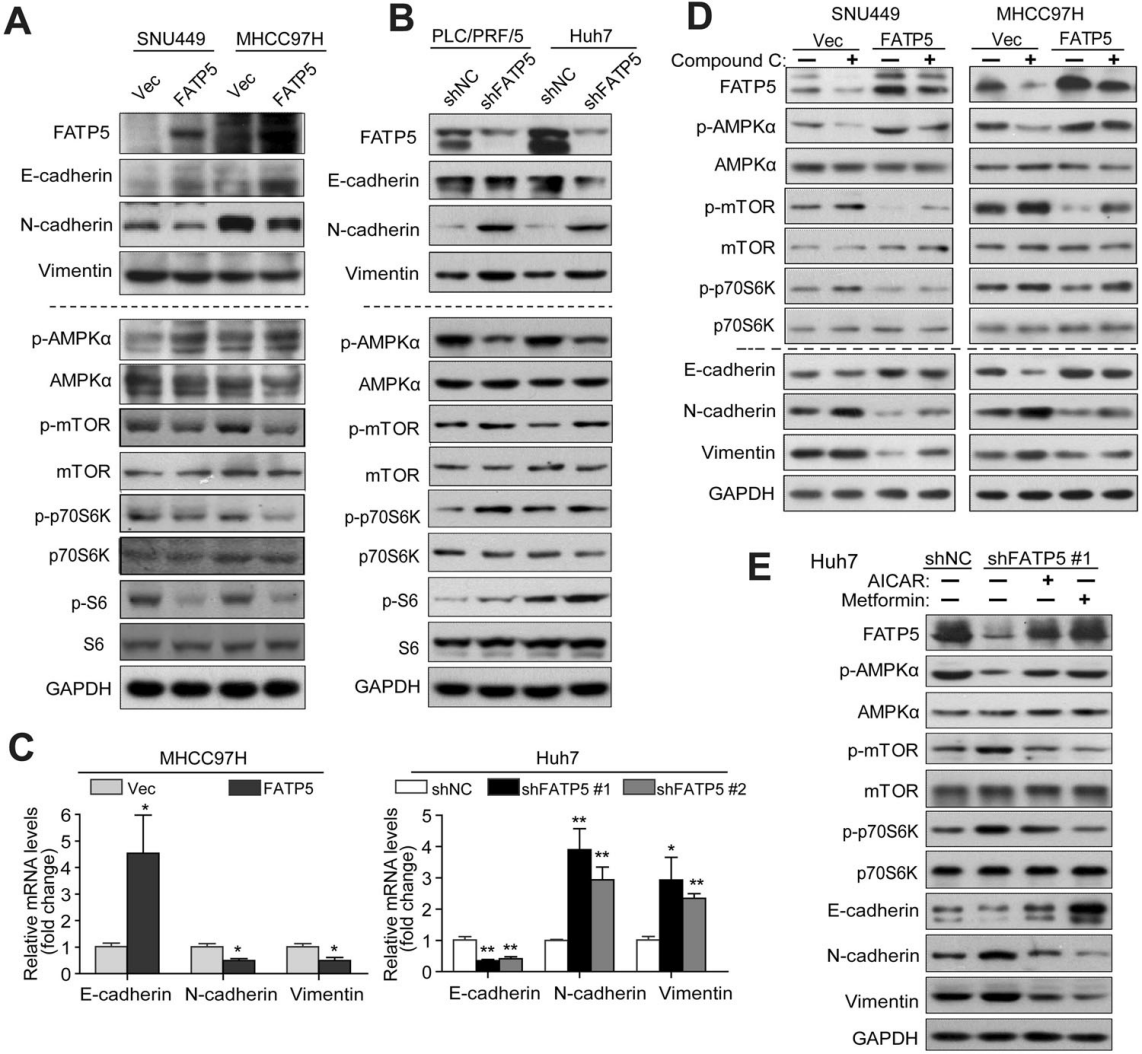
FATP5 inhibits epithelial-to-mesenchymal (EMT) by regulating AMPK-mTOR-S6K signaling. **A**, **B** Expression of EMT markers (E-cadherin, N-cadherin, and vimentin) and phosphorylation status of AMPKα, mTOR, S6K, and S6 in FATP5-overexpressing cells (SNU449 and MHCC97H transfecting Vec or FATP5) or FATP5 knockdown HCC cells (PLC/PRF/5 and Huh7 cells expressing shNC or shFATP5 #1) were analyzed by western blotting. **C** The mRNA levels of E-cadherin, N-cadherin, and vimentin in indicated HCC cells transfected with empty vector or FATP5, or infected with LV-shNC or LV-shFATP5 (sequence #1 and #2) were evaluated by qRT-PCR. All mRNA values was normalized to β-actin (ΔCt) and compared with that in their corresponding control cells (ΔΔCt). The fold change was calculated and presented as 2^−△△Ct^, and the expression levels in MHCC97H-Vec or Huh7-shNC was set as 1. ^*^P < 0.05 and ^**^P < 0.01 (two-tailed *t*-test). **D**, **E** SNU449 and MHCC97H-Vec and FATP5 cells were treated with or without Compound C (10 μM) for 48 h (**D**). Huh7-shNC and shFATP5 #1 cells were treated with or without AICAR (1.5 mM) or metformin (5 mM) for 48 h (**E**). Expression of EMT markers and phosphorylation status of AMPK-mTOR-S6K signaling were analyzed by western blotting. For all experiments in (**A**, **B**, **D**, **E**), GAPDH was used as internal loading control. Abbreviations: p-, phosphorylated.

To explore the underlying mechanisms of FATP5-mediated suppression of HCC metastasis, we first investigated whether FATP5 regulates the EMT program, which is a hallmark and crucial cellular process for tumor metastasis [17], by detecting the expression levels of EMT-related markers. Western blotting indicated that FATP5 upregulated E-cadherin, the most critical epithelial marker, and inhibited the expression of mesenchymal markers, including N-cadherin and vimentin, while FATP5 silencing led to the opposite influence on these EMT factors (Fig. 3A, B). Consistently, the mRNA level of E-cadherin was also decreased, but N-cadherin and vimentin were elevated in FATP5-deficient HCC cells compared with that in shNC-transfected cells (Fig. 3C). Hence, the EMT process is involved in FATP5-induced suppression of HCC cell metastasis.

Previous studies have reported a negative correlation between the EMT process and AMPK signaling in several malignancies [18–20]. Additionally, activation of AMPK is associated with a favorable prognosis and exerts anticancer effects by inhibiting mTOR signaling and other metastasis-related factors [19, 21]. Therefore, we considered whether FATP5 regulates AMPK-mTOR signaling to influence the EMT process and HCC cell metastasis. We first detected whether FATP5 interacted with AMPK or mTOR directly. Co-immunoprecipitation assays confirmed no direct interaction between FATP5 and AMPK or mTOR, suggesting an indirect role of FATP5 in connection with the AMPK-mTOR pathway (Supplementary Fig. 3D). FATP5 overexpression induced AMPK activation and subsequently inhibited the phosphorylation of mTOR and its downstream ribosomal proteins S6 kinase (p70 S6K) and S6 in SNU449 and MHCC97H cells, while FATP5 depletion weakened the phosphorylation status of AMPK and promoted mTOR-S6K-S6 signaling activation, with total AMPK and mTOR protein expression almost unchanged (Fig. 3A, B). Furthermore, AMPK inhibitor Compound C treatment rescued FATP5-overexpression-mediated suppression of mTOR, S6K, N-cadherin, and Vimentin in HCC cells (Fig. 3D), while activating AMPK by metformin, a widely accepted drug against type 2 diabetes, blocked FATP5-deficiency-induced phosphorylation of the mTOR-S6K pathway and decreased the high levels of mesenchymal markers in Huh7 cells (Fig. 3E). These results supported that the suppressive effect of FATP5 on EMT was largely dependent on AMPK activation and mTOR-S6K inactivation. Our data suggested that a signaling network involving AMPK, mTOR, S6K and S6 may play important roles in FATP5-deficiency-induced EMT and HCC metastasis.

### FATP5 induces the activation of AMPK by reprogramming glycolysis and energy metabolism of HCC cells

**Fig. 4:**
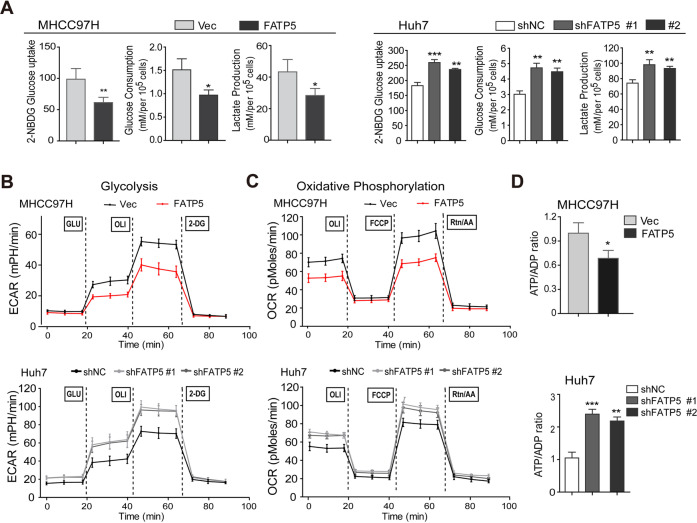
FATP5 induces the activation of AMPK by reprogramming glycolysis and energy metabolism of HCC cells. **A** HCC cells were incubated with 2-NBDG for 30 min and then washed and re-suspended with DMEM medium. The cellular fluorescence intensities were measured by flow cytometry. Glucose and lactate concentrations in normal culture medium and supernatants after cell incubation for 48 h were detected to calculate the glucose consumption and lactate production. **B**, **C** Both extracellular acidification rates (ECAR) and oxygen consumption rates (OCR) in different time points were measured using a Seahorse XF96 Extracellular Flux Analyzer either under basal conditions or after treatment with according inhibitors. Representative curves were shown and each data was presented as mean ± SD, with the average value of 6 independent wells. **D** The cellular ATP content in HCC cells was measured and the ATP/ADP ratio was expressed as the fold change relative to their corresponding Vec- or shNC-expressing cells (presented as equal to 1). All results in (**A**) and (**D**) were normalized to cell numbers and protein contents, respectively, ^*^P < 0.05, ^**^P < 0.01 and ^***^P < 0.001 vs. negative controls. Abbreviations: OLI, oligomycin; FCCP, carbonyl cyanide p-trifluoromethoxy-phenylhydrazone; Rtn/AA, rotenone/antimycin; 2-DG, 2-deoxyglucose.

Because AMPK is a critical energy regulator that senses the ATP content and is activated once cellular ATP is depleted [22], we further elucidated whether FATP5 manipulates the AMPK/mTOR signaling in an ATP-dependent manner or because of other FATP5-driven metabolic reprogramming. To address this issue, we first explored the impact of FATP5 on cellular metabolic phenotypes and energy production in HCC cells. Glucose-dependent metabolic rewiring, also known as the Warburg effect, is the most common feature of human cancers, with characteristics of enhanced glycolysis that converts most glucose to lactate for energy production [23]. Exogenous expression of FATP5 remarkably attenuated the glucose uptake capacity and reduced cellular glucose consumption and lactate production in culture medium, while silencing FATP5 abolished these effects (Fig. 4A). We next validated these FATP5-driven metabolic phenotypes in HCC cells using a Seahorse XF96 Extracellular Flux Analyzer. Consistently, FATP5-expressing cells displayed a decreased extracellular acidification rate (ECAR), indicating impaired glycolytic capacity, compared with their counterparts, while the loss of FATP5 had an inverse impact on ECAR in Huh7 (Fig. 4B and Supplementary Fig. 4A). Considering that the excess metabolites of glycolysis are transported into mitochondria for mitochondrial oxidative phosphorylation (OXPHOS), we also evaluated the effect of FATP5 on this major metabolic process. FATP5-overexpressing cells exhibited reduced basal and maximal respiration capacities, while FATP5 knockdown enhanced mitochondrial OXPHOS, with an elevated oxygen consumption rate (OCR) at both basal and maximal levels (Fig. 4C and Supplementary Fig. 4B). Notably, an increased rate of ECAR/OCR was found in FATP5-deficient HCC cells, indicating a metabolic switch toward the glycolytic pathway, probably for energy supply. Because the cellular ATP content can reflect the level of energy metabolism, we further detected the cellular ATP level and noted that FATP5-overexpressing cells produced less ATP than control cells, while FATP5 depletion markedly enhanced ATP production (Fig. 4D). Collectively, our metabolic analyses implied that FATP5 loss augments glucose flux to support the glycolytic pathway and induce ATP production, inhibiting AMPK phosphorylation and activating mTOR signaling to promote EMT and cancer cell metastasis.

### Blocking AMPK rescues FATP5-induced inhibition of EMT and metastasis in HCC cells

**Fig. 5:**
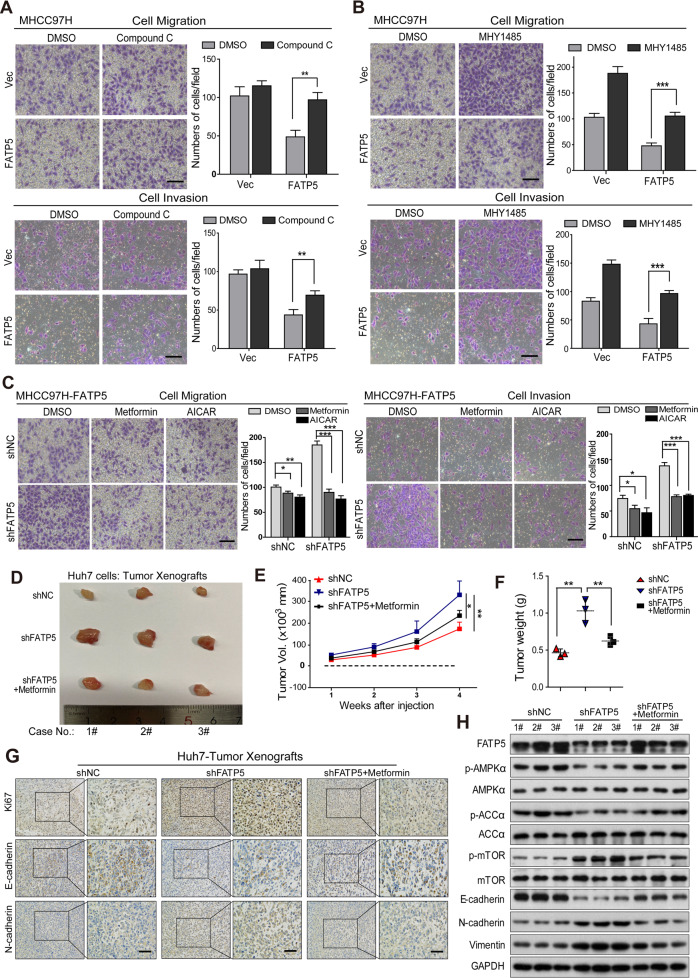
Activation of AMPK suppresses the EMT process in HCC cells and exhibits antitumor effects in FATP5-deficient xenografts. **A**–**C** MHCC97H cells stably expressing Vec or FATP5 were stimulated with or without Compound C (10 μM) (**A**) or MHY1485 (10 μM) (**B**) for 48 h. MHCC97H-FATP5 cells infecting with LV-shNC or LV-shFATP5 #1 were treated with metformin (5 mM) or AICAR (1.5 mM) for 48 h (**C**). Then the migration and Matrigel invasion assays were performed to measure the migratory and invasive capacities of above indicated HCC cells. The migrated or invaded cells were microscopically counted, and data were presented as the average number of cells from 5 independent microscopic fields. Scale bar: 50 μm. All values in (**A**–**C**) expressed as mean ± SD, ^*^P < 0.05, ^**^P < 0.01, and ^***^P < 0.001. **D**. Mice injected with 1.5 × 10^6^ Huh7 cells expressing shNC or shFATP5 were treated with control vehicle or daily 4 mg/ml metformin in drinking water for 3 weeks. **E**, **F** Xenograft growth conditions from each group (*n* = 3) were evaluated at indicated time points, and tumor weights were detected after mice were sacrificed. All results were presented as mean ± SD, ^*^P < 0.05 and ^**^P < 0.01. **G** IHC analyses of Ki67, E-cadherin, N-cadherin in Huh7 shNC or shFATP5-expressing xenografts after treatment of metformin, and representative photographs were shown. Scale bar: 50 μm. **H** Western blot analyses of the expressions of EMT markers and phosphorylation status of AMPK-mTOR signaling in harvested Huh7-derived tumors as mentioned above. GAPDH was used as internal loading control. #1–3 represented the number of mice in different treated groups.

We next confirmed the functional roles of AMPK/mTOR signaling in FATP5-mediated suppression of HCC cell migration and invasion using a specific AMPK inhibitor, Compound C, and an mTOR activator, MHY1485. Transwell assays revealed that either Compound C or MHY1485 treatment substantially abrogated the inhibitory effects of FATP5 on the cell motility and invasiveness of HCC cells (Fig. 5A, B and Supplementary Fig. 5A, B). Similarly, knockdown of AMPK by shRNA significantly restored the cell migratory and invasive capacities of FATP5-expressing HCC cells (Supplementary Fig. 5C, D). Conversely, AMPK activation using metformin and another AMPK-specific activator, AICAR, significantly attenuated the cell migratory and invasive capacities in FATP5-deficient cells (Fig. 5C). In summary, these data revealed that FATP5 opposes HCC invasiveness by promoting AMPK activity and suppressing mTOR signaling.

### Activation of AMPK suppresses the EMT process and exhibits antitumor effects in FATP5-deficient xenografts

We next extended our investigations to xenograft models using Huh7 cells expressing shNC or shFATP5 and then treated them with or without the AMPK activator metformin. As expected, FATP5 knockdown significantly promoted HCC tumorigenesis, as revealed by a higher tumor growth rate and a larger maximum tumor size, whereas AMPK activation by metformin reduced the tumor burden of mice in the Huh7-shFATP5 group (Fig. 5D–F). These data demonstrated that FATP5 depletion enhances the tumorigenic capacity of HCC cells in vivo, while activation of the AMPK pathway by metformin abolishes these effects, implying an essential role of AMPK in regulating FATP5-mediated inhibition of HCC progression and metastasis.

Additionally, IHC analyses showed that the knockdown of FATP5 promoted cell proliferation, suppressed cell apoptosis, and induced the EMT process by regulating E-cadherin and N-cadherin expression during HCC tumorigenesis (Fig. 5G and Supplementary Fig. 6). Consistently, silencing FATP5 led to decreased AMPK activity and enhanced mTOR-S6K signaling in tumor samples, while stimulating the AMPK pathway by metformin treatment reversed these changes (Fig. 5H). Taken together, our findings revealed that loss of FATP5 confers a growth advantage and metastatic potential in HCC cells by regulating the AMPK/mTOR pathway in vivo. Thus, targeting AMPK/mTOR signaling may represent an efficient treatment strategy for FATP5-deficient HCCs.

## Discussion

In addition to the widely accepted aerobic glycolysis, exacerbated de novo lipogenesis has been proposed as an emerging metabolic hallmark of HCC that is critical for cancer cell growth and survival under stressful conditions [8, 24]. Intracellular FAs are required to generate cellular membranes, signaling molecules and energy storage [5]. In addition to the de novo synthesis of FAs, the uptake and transport of exogenous FAs from the external environment is a critical alternative aspect of metabolic abnormalities but has received less attention, particularly in HCC tumorigenesis. Although previous evidence has revealed contrasting roles of FA transport in distinct cancer types, HCC cells prefers de novo biosynthesis of FAs instead of acquiring FAs from exogenous sources. The aforementioned observations suggested the inhibitory role of FA transport in HCC development, yet the underlying mechanisms involved in this metabolic phenotype remain elusive.

Metabolic changes such as FA metabolism are mainly characterized by alternations in the expression and activity of key enzymes that catalyze the committed steps [5, 25]. Encoded by the solute carrier family 27 member 5 gene (*SLC27A5*), FATP5 is a major FA transporter that is exclusively detected in liver tissues [10]. FATP5 silencing has been recently reported to maintain cellular redox homeostasis and thus support HCC proliferation and progression [15]. Consistent with these findings, our results also confirmed that FATP5 was highly expressed in normal liver tissues but weakly detected in HCC cell lines and HCC samples. Interestingly, the expression of FATP5 was barely detected in PVTT and much lower than that in HCC tissues, suggesting a closer relevance between FATP5 and invasive tumor features. Additionally, patients with FATP5-negative tumors frequently had more aggressive phenotypes and poorer clinical outcomes, and FATP5 loss was identified as an independent risk factor associated with tumor recurrence and survival. A recent study has suggested that the methylation level of FATP5 promoter was significantly increased, thus leading to FATP5 downregulation in HCC tissues [15]. However, whether there exists other critical regulators of FATP5 deficiency in HCC is not involved in the present study, which requires further investigation.

**Fig. 6:**
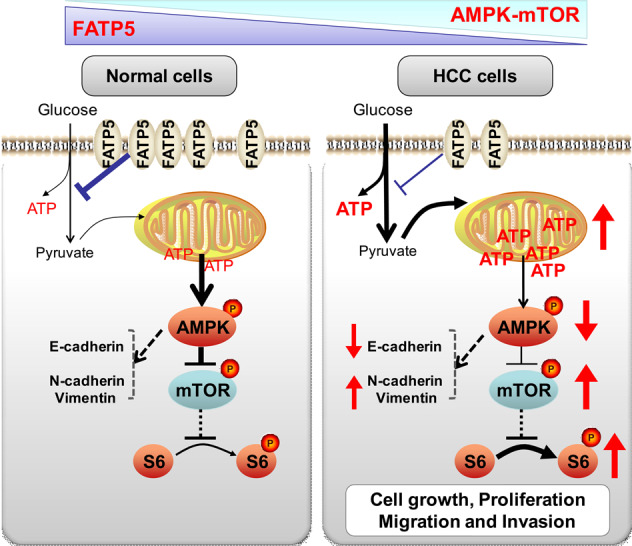
Proposed working model of FATP5-mediated effects on HCC cells. The loss of FATP5 leads to an increase in metabolic flux of glycolysis and ATP generation, which inhibits AMPK and activates its downstream mTOR-S6K-S6 signaling pathway, thereby promoting HCC cell growth, migration, invasion, and HCC tumorigenesis. Abbreviations: p-, phosphorylated.

Based on loss- and gain-of-function experiments in vitro, FATP5 was proven to be a regulator of cellular metabolic pathways as well as a potent inhibitor of the EMT process. Further investigation of the mechanism indicated that FATP5 suppresses HCC invasion and metastasis by modulating the AMPK/mTOR/S6K pathway by reprogramming cellular energy metabolism (Fig. 6). Additionally, experimental animal models in vivo confirmed that the loss of FATP5 promotes HCC tumorigenesis and metastasis via the AMPK pathway, while activating AMPK by metformin reversed these effects and dramatically suppressed tumor progression. Collectively, our findings demonstrate an anti-metastatic role of FATP5 in HCC and provide a promising prognostic predictor for HCC recurrence and survival.

Cancer metastasis is a complex process including cell detachment from the primary tumor, circulation of migrated cells through blood vessels, and formation of secondary tumor foci after reaching distant organs [26]. During this period, cancer cells lose their cell-cell contacts and escape from the primary tumor site by acquiring mesenchymal features through the EMT process, which promotes the migration and invasion of cancer cells [27]. EMT is characterized by the loss of epithelial markers, such as E-cadherin, and enhanced expression of mesenchymal markers, including N-cadherin and vimentin. Therefore, we investigated whether the EMT process was involved in FATP5-mediated cell metastasis. As expected, our results demonstrate that FATP5 depletion decreases E-cadherin while increasing N-cadherin and vimentin at both the protein and mRNA levels. Similar to the alterations in EMT-related markers, the capacities of cell migration and invasion were also decreased in FATP5-expressing HCC cells.

We next explored the specific mechanisms by which FATP5 modulates the EMT process in HCC. The AMPK pathway is required for EMT induction [28, 29], while AMPK activation inhibits the EMT process in different cancer types [18–20]. Considering this evidence, we asked whether the AMPK pathway is implicated in the FATP5-mediated EMT process and HCC cell metastasis. Western blotting showed that FATP5 knockdown inhibited AMPK activity and enhanced mTOR activation, thereby supporting the EMT process. Furthermore, metformin administration reversed FATP5-silencing-induced promotion of EMT by activating AMPK. Another AMPK activator, AICAR, also achieved the same effect on the FATP5-mediated EMT program. Thus, our data clearly illustrated that the FATP5-meidated EMT process is AMPK pathway dependent, and intervention in AMPK signaling may play an essential role in preventing tumor metastasis in low-FATP5-expressing patients. Notably, no direct physical interaction between FATP5 and AMPK or mTOR was observed in HCC cells, supporting that FATP5 may indirectly activate the AMPK/mTOR pathway by manipulating cellular glycolysis and energy production.

AMPK is a crucial metabolic energy sensor controlling both normal and malignant cell metabolism [30]. Once activated by decreased cellular ATP/AMP ratio, AMPK stimulates catabolic pathways to maintain a sufficient energy supply and negatively regulates its downstream effector mTOR, thus modulating cancer cell proliferation, invasion and metastasis [31]. Dysregulation of the AMPK/mTOR pathway has been widely reported in various cancers, and AMPK inactivation is also closely associated with aggressive malignant behaviors and serves as a promising therapeutic target [32]. Our metabolic analyses demonstrated that enforced FATP5 decreases ATP generation in HCC cells to activate AMPK and maintain cellular AMP/ATP levels. At the same time, FATP5 overexpression is related to HCC cell invasion and metastasis and vice versa. Thus, in HCC cells, FATP5 loss may increase both glycolysis and the energy metabolism pathway, leading to inhibition of AMPK and activation of mTOR, thereby accelerating HCC cell migration and invasion both in vitro and in vivo. This work describes for the first time the involvement of AMPK/mTOR signaling pathway in regulating the FATP5-mediated EMT process and HCC progression via metabolic reprogramming. However, other possible mechanisms by which FATP5 regulates AMPK pathway and subsequently consequences of AMPK activation are not involved, which still need to be addressed further.

Previous studies have pointed out that AMPK activation is strongly associated with a better survival rate and favorable clinical outcomes in several malignancies [19, 21]. In this setting, multiple pharmacological activators, such as metformin, phenformin, A769662, and AICAR, have been confirmed to cause AMPK activation and inhibit tumorigenesis in different animal experimental models [33–35]. Other clinical evidence also showed that metformin treatment reduces the risk of HCC development in patients with type 2 diabetes mellitus, implying a protective role of metformin on HCC initiation and progression [36]. In this study, metformin, a commonly used antidiabetic drug, and AICAR were used to modulate AMPK activity and exerted inhibitory roles in HCC cell metastasis by activating AMPK when FATP5 was depleted. By contrast, blockade of AMPK by Compound C or shRNA, as well as activation of mTOR by the specific activator MHY1485, significantly reversed the inhibitory effects of FATP5 on cancer cell metastasis. These results further confirmed the anticancer role of metformin as a useful regulator and a novel therapeutic option for HCC management by activating AMPK. Meanwhile, considering the huge heterogeneity and lack of effective tumor biomarkers in HCCs, the expression levels of FATP5 may be identified as a novel metabolic marker to distinguish different subtypes of HCCs. Thus, for patients with FATP5-deficient HCC, metformin treatment may provide a substantial clinical benefit as a desirable adjuvant anti-HCC therapy.

In summary, this study elucidated that FATP5 blocks the migration and invasion of HCC cells by inhibiting the EMT process, while its loss promoted tumor progression in part by regulating cellular glycolysis and energy production. These events lead to AMPK inhibition and mTOR/S6K signaling activation to promote cell metastasis. These findings have expanded our understanding of FATP5 in HCC progression and have shed new light on the development of potential therapeutic candidates for HCC treatment in clinical practice. More specifically, activation of AMPK may serve as an effective target to prevent HCC metastasis, particularly for patients with low-FATP5-expressing HCCs.

## Materials and methods

### Patients and samples

128 cases of HCC specimens and matched nontumoral adjacent tissues were obtained from patients who underwent curative resection for pathologically diagnosed HCC in the Eastern Hepatobiliary Surgery Hospital (Shanghai, China) and were used for the TMA. Additional 42 paired fresh HCC samples, including 18 cases of primary HCC with PVTT, were used for qRT-PCR, western blotting, or IHC analyses. This study was approved by research ethical committee of the Eastern Hepatobiliary Surgery Hospital, with all enrolled patients had written informed consent.

The gene expression data were obtained from the TCGA and GEO databases using appropriate key words (Supplementary Table 4). A set of microarray data from the ONCOMINE databases (http://www.oncomine.org) were collected using filtering criteria of “*SLC27A5*”, “Hepatocellular carcinoma and Normal” and “mRNA data type”. Then a meta-analysis was performed to compare the expression levels of FATP5 (*SLC27A5*) between HCC and matched nontumoral tissues.

### Cell cultures, gene overexpression, and RNA interference

Human HCC cell lines were obtained from Cell Bank of Type Culture Collection of the Chinese Academy of Sciences (Shanghai, China) and were routinely cultured in Dulbecco’s modified Eagle’s medium (DMEM) supplemented with 10% fetal bovine serum (FBS) (Gibco, Invitrogen, MA, USA) and 1% Penicillin-Streptomycin at 37 °C in 5% CO_2_. Compound C and MHY1485 were obtained from MedChem Express (Monmouth Junction, NJ, USA). Metformin and 5-aminoimidazole-4-carboxamide 1-β-D-ribofuranoside (AICAR) were purchased from Sigma-Aldrich (St Louis, MO, USA).

For establishment of stable cell lines, full-length cDNA of human *SLC27A5* (FATP5) was amplified by PCR using designed primers (Forward: ATTTCCGGTGAATTCATGGGTGTCAGGCAACAGTT; Reverse: AGAGGGGGCGGCCGCTGAGAGCCTCCAGGTTCCCT), and the purified product was cloned into the *EcoR* I/*Not* I sites of pLVX-mCMV-ZsGreen1-puro vector (Asia-Vector Biotechnology Co. LTD, Shanghai) according to the manufacturer’s instructions. SNU449 and MHCC97H cells were transfected with recombined vector using Lipofectamine 2000 reagent (Invitrogen, Carlsbad, CA) and then selected by puromycin (Sigma-Aldrich, St. Louis, USA) after 48 h post-transfection. Cells transfected with pLVX-empty vector were used as controls. For RNA interference, the shRNAs for FATP5 were designed and inserted into the *Xho* I/*MIu* I sites of PGIPZ lentivirus vector (Asia-Vector Biotechnology Co. LTD, Shanghai). Lentivirus products were generated and transfected into HCC cell lines in the presence of polybrene for 6 h. After 1 day post-infection, the supernatant was removed and stable clones were selected by puromycin for another 48 h. The sequences for shRNA are listed in Supplementary Table 5. The lentiviral particles containing AMPKα1 shRNA (Santa Cruz, CA; sc-29673-V) were added to HCC cell for 48 h and puromycin (1 μg/ml) was used to select stable shAMPKα1-expressing cells.

### Metabolic assays

All the measurements of metabolism-related indicators were performed as previously reported [37]. Briefly, the lactate production and glucose consumption in culture medium were detected using EnzyChrom™ D-Lactate Assay Kit (Bioassay, CA, USA) and BS-200 Chemistry Analyzer (Mindray, China). Cell numbers were counted to adjust the above results. Intracellular ATP levels were determined using ATP Bioluminescence Assay Kit (Beyotime, Nanjing, China) and the concentrations were normalized to protein content in each sample. For glucose uptake assay, a deoxyglucose analog 2-deoxy-2-[(7-nitro-2,1,3- benzoxadiazol-4-yl)amino]-D-glucose (2-NBDG; 10 μM) (Life Technologies, USA) was added in culture medium and the fluorenscence intensities of labeled HCC cells were analyzed using flow cytometry. For energy metabolism assays, Seahorse XF96 extracellular flux analyzer was used to measure OXPHOS by analyzing the OCR, and measure glycolysis on the basis of the ECAR. Briefly, 2 × 10^4^ HCC cells were seeded into XF96-well plates (Seahorse Bioscience, North Billerica, MA, USA) per well and maintained in serum-free DMEM medium for 12 h. After equilibration with unbuffered DMEM for pH stabilization, the prepared cells were subjected to measure the basal and maximum metabolic flux rates, especially after treatment with 5 μM glycolysis inhibitor, 2-deoxyglucose (2-DG), and mitochondrial poisons including 1 μM oligomycin (OLI), 1 μM fluorocarbonyl cyanide phenylhydrazone (FCCP), and 1 μM rotenone/antimycin A (Rtn/AA) at indicated time points.

### In vitro cell-behavior assays

Based on the standard procedures, wound-healing assay, cell migration and invasion assays were conducted to determine the functional roles of FATP5 in vitro. HCC cells were seeded in 12-well plates and then maintained in serum-free DMEM medium once the cell monolayer was formed. The scraping wound was created with a 200 μL pipette tip along the bottom after cell adhesion, and the wound closure was photographed and measured at 48 h using an inverted light microscope (Olympus, Tokyo, Japan). Cell migration and invasion assays were conducted using 8 mm pore size transwell filter chambers (Costar, Corning, NY, USA) and Matrigel Invasion Chambers (BD Biosciences, CA, USA), with re-suspended HCC cells and FBS-free medium placing into the upper chamber while 10% FBS medium adding to the lower chamber. After incubation for 48 h, the invaded cells adhering to the lower surface of membrane were fixed and stained with crystal violet dye, and then captured and counted in 6 random fields using the microscope.

### In vivo xenograft assays

First, 6–8-week-old male BALB/c nude mice (20–23 g) were purchased from experimental animal center of Chinese Science Academy (Shanghai, China). For tumorigenic assay in vivo, 1.5 × 10^6^ Huh7-shFATP5 (#1) or control cells (shNC) were subcutaneously planted in the right dorsal flank of each mouse. At 1 week after implantation, the randomly selected mice injected with shFATP5 HCC cells were daily administered with metformin at a dilution of 4 mg/ml in the drinking water or vehicle as untreated control for another 3 weeks. The tumor growth condition was observed more than 4 weeks, and the tumor volume was calculated weekly according to a formula *V* = 0.5 × *L* × *W*^2^ (*V*: volume; *L*: length; *W*: width) until sacrificed. Then the xenografts were weighted and the tumor tissues were homogenized into protein lysate for western blotting, or promptly fixed in paraffin overnight for IHC staining (DAKO, Denmark, EU). In the pulmonary metastatic model, 1 × 10^6^ MHCC97H-Vec and FATP5 cells were injected into the caudal veins of 6-week-old nude mice and survival condition was recorded until 80 days after injection. After mice were scarified at 8–10 weeks, the number and maximum size of metastatic foci in lung was counted and compared. All animal experiments were performed in accordance with the Second Military University Animal Care Facility and the National Institutes of Health guidelines.

### Statistical analysis

All the continuous data were presented as mean ± standard deviation (SD). Differences between two groups were analyzed by two-tailed Student’s *t*-test. Correlations between FATP5 expression and clinicopathological features were assessed by Pearson *χ*2-test or the Fisher’s exact test. OS was calculated from the date of surgery to final follow-up or death, whereas DFS was defined as the interval between the dates of surgery and tumor recurrence or last follow-up. Kaplan-Meier analyses were performed to plot survival curves, and log-rank test was used for comparison of survival rates. The Cox multivariate hazard regression model was established to identify risk factors associated with survival and recurrence. All measurements were repeated at least three times, and all statistical analyses were performed using SPSS 21.0 software (IBM Corporation, Armonk, New York, USA). A value of *P* < 0.05 was considered statistically significant.

More detailed materials and methods are available in the Supplementary Information.

### Role of sponsor

The funding sources had no role in the design and conduct of the study; collection, management, analysis, and interpretation of the data; preparation, review, or approval of the manuscript; and decision to submit the paper for publication.

## Supplementary information

Supplementary file.
