# Supplementary material for: Fatty acid transport protein-5 (FATP5) deficiency enhances hepatocellular carcinoma progression and metastasis by reprogramming cellular energy metabolism and regulating the AMPK-mTOR signaling pathway
Source: Oncogenesis. 2021 Nov 12;10(11):74. doi: 10.1038/s41389-021-00364-5 (PMC8589992; doi:10.1038/s41389-021-00364-5)
Supplement: Supplementary file 1 — Supplementary file. [file 41389_2021_364_MOESM1_ESM.docx]

**Supplementary Materials and Methods**

**Specimens and Patient Follow-up**

All fresh HCC and their corresponding peri-tumoral tissues were collected promptly in operation room once the samples were resected, and then maintained on ice. For western blot analysis, tissues were mechanically grinded and lysed in RIPA buffer to harvest the total proteins. For Tissue Microarrays (TMAs) and Immunohistochemical (IHC) assays, HCC samples were fixed in paraffin and then embedded into wax blocks until use. TMAs were constructed by Shanghai Biochip Co, Ltd, Shanghai, China. A total of 128 consecutive HCC patients who underwent curative-intent liver resection between January 2010 and November 2012 at our hospital were used to prepare TMAs and their chinicopathological characteristics were retrospectively reviewed. Both the inclusion criteria and follow-up protocol of all patients were described in our previous study^(^[^1^](#_ENREF_1)^)^. Briefly, patients were prospectively followed up with physical examination, serum α-fetoprotein (AFP), ultrasonography or contrast-enhanced computed tomography (CT) scan or magnetic resonance imaging (MRI) of the abdomen, and chest X-ray once every 2 months for the first 6 months after hospital discharge, and then every 3 months for the next 1.5 years. For patients who were free of HCC recurrence 2 years after surgery, recurrence surveillance was performed at a 6-month interval thereafter. Tumor recurrence was suspected based on appearance of new intra- or extra-hepatic tumor lesion(s) that possessed typical imaging characteristics consistent with HCC on contrast-enhanced CT or MRI, with or without an elevation of serum AFP level. The follow-up was finished on March 2016.

**RNA Extraction and Real-time PCR**

Total RNA was extracted from cultured HCC cells or clinical specimens using TRIzol reagent (Invitrogen) and then reverse-transcribed into cDNA using the M-MLV Reverse Transcriptase (Invitrogen), according to the manufacturer’s instructions. Quantitative real-time PCR was conducted using an ABI 7300 Fast Real-Time PCR System (Applied Biosystems, Foster City, CA) and SYBR Green PCR kit (Applied TaKaRa, Otsu, Shiga, Japan) with specific primers (Supplementary Table 6). The expression of GAPDH was used as an endogenous control to normalize the RNA level. The relative expression of target gene was calculated using the ΔCt method (-ΔΔCt), or presented as “fold change” by normalizing the expression level detected in corresponding control cells or non-tumoral tissues.

**Western Blotting**

Western blotting was conducted as we previously described^(^[^2^](#_ENREF_2)^)^. Briefly, cultured cells or clinical tissues were lysed in RIPA Buffer (Beyotime Biotechnology, Jiangsu, China) and then centrifuged at 12000×g for 15 min to harvest protein extracts. Specific primary antibodies used in this study are listed in Supplementary Table 7. After incubating with indicated primary antibodies, the immunocomplexes were probed with anti-rabbit horseradish peroxidase (HRP)-conjugated IgG antibodies (1:5000 dilution; cat No. 7074T; Cell Signaling Technology, MA, USA), following the detection by enhanced chemiluminescence (ECL) reagent (Millipore, CA, USA). All images were obtained and processed using an Odyssey fluorescence scanner system (Li-Cor, Lincoln, NE).

**Expression Constructs and Transfection**

Full-length cDNAs for human AMPKa1, mTOR, and FATP5 expression plasmids were amplified by RT-PCR using designed primers as presented below. The purified cDNA products were blunted and cloned in-frame into an N-terminal HA, Myc or FLAG expression pcDNA3.3 vector (Invitrogen) at BamH I/Xho I sites according to the manufacturer’s instructions.

For plasmids transfection, routinely cultured HEK-293T cells were maintained in 10-cm culture plates at a confluency of 70% and then transfected with indicated expression constructs using Lipofectamine 2000 transfection reagent (Invitrogen). After 8 hours post-transfection, the supernatants were replaced with normal culture medium and cells were harvested at 48 hours after plasmids transfection.

AMPKa1-Forward: AGAGAATTCGGATCCATGCGCAGACTCAGTTCCTGGA,

AMPKa1-Reverse: CTTCCATGGCTCGAGTTATTGTGCAAGAATTTTAATTA;

mTOR-Forward: AGAGAATTCGGATCCATGCTTGGAACCGGACCTGC,

mTOR-Reverse: CTTCCATGGCTCGAGTTACCAGAAAGGGCACCAGCC;

FATP5-Forward: AGAGAATTCGGATCCATGGGTGTCAGGCAACAGTT,

FATP5-Reverse: CTTCCATGGCTCGAGTCAGAGCCTCCAGGTTCCCT.

**Co-immunoprecipitation (co-IP)**

Cell lysate was harvested and protein concentration was determined as described in western blotting. A total of 500 μg protein lysate was diluted in 1 ml lysis buffer and incubated with HA-tagged antibody or anti-IgG (negative control, 2 μg, Abcam, ab200699) at 4℃ on a rotator overnight. Then the immunoprecipitation samples were mixed with 50 μl Pierce Protein A/G-conjugated Magnetic Beads (Thermo Fisher Scientific, USA, #88803) and rotated for 2 hours. Afterwards, immunoprecipitates were centrifugated at 2500 rpm and washed three times with Wash Buffer, followed by heating and re-suspension in 1 × SDS-PAGE. Finally, the supernants were harvested and subjected to western blotting.

**IHC Staining**

IHC staining was conducted according to standard protocols. Briefly, paraffin-fixed sections of clinical specimens or TMA slides were incubated with rabbit polyclonal anti-human FATP5 (1:200 dilution, cat. No. ab224069; Abcam, Cambridge, UK) at 4°C overnight, following probed with corresponding secondary anti-rabbit IgG (ZSGB-BIO, Beijing, China). Finally, 3, 3’-diaminobenzidine (DAB) reagent (Dako, Carpinteria, CA) was applied to envisage reaction products for visualization followed by hematoxylin counterstaining. The stained slides were scanned and images were processed using Pannoramic Viewer System or Aperio Scan-Scope software (Media Cybernetics, Inc., Bethesda, MD). The staining conditions, which represented the expression levels of target genes, were assessed by the Aperio Image-scope software according to the staining intensities of specific areas and the percentage of positive-staining cells, as we previously reported^(^[^2^](#_ENREF_2)^)^. For Ki67, E-cadherin, and N-cadherin Staining, the Dako antibody detection system was also used as mentioned above.

**Apoptosis Assay**

The Annexin V-FITC apoptosis detection kit (Invitrogen) was used for *in vitro* apoptosis assay as previously reported^(^[^3^](#_ENREF_3)^)^. Briefly, HCC cells were collected and re-suspended in binding buffer with Annexin V and propidium iodide (PI) for 30 min in dark, and then Annexin V staining was conducted by flow cytometry within 1 hour according to the manufacturer’s protocols. The apoptotic rates were analyzed and defined as the total proportion of cells at early (Annexin V-positive and PI-negative) and late (Annexin V/PI-double positive) stage of apoptosis.

**References**

1. Wang MD, Li C, Liang L, Xing H, Sun LY, Quan B, et al. Early and Late Recurrence of Hepatitis B Virus-Associated Hepatocellular Carcinoma. The oncologist. 2020. Epub 2020/05/31.

2. Wang MD, Wu H, Fu GB, Zhang HL, Zhou X, Tang L, et al. Acetyl-coenzyme A carboxylase alpha promotion of glucose-mediated fatty acid synthesis enhances survival of hepatocellular carcinoma in mice and patients. Hepatology. 2016;63(4):1272-86. Epub 2015/12/25.

3. Chen S, Cheng AC, Wang MS, Peng X. Detection of apoptosis induced by new type gosling viral enteritis virus in vitro through fluorescein annexin V-FITC/PI double labeling. World journal of gastroenterology. 2008;14(14):2174-8. Epub 2008/04/15.

**Supplementary Figures and Figure Legends**

**Supplementary Figure 1. (Related to Figure 1)**

Relative mRNA expression levels of *SLC27A5* (FATP5) in HCC and non-tumor liver tissues from four independent analyses published in ONCOMINE database (*Roessler Liver*, Cancer Res, 2010; *Roessler Liver 2*, Cancer Res, 2010; *Chen Liver*, Mol Biol Cell, 2002; and *Wurmbach Liver*, Hepatology, 2007). Values represented as the mean ± SD. The log2 median-centered intensity was calculated to compare the expression differences (two-tailed *t*-test).

**Supplementary Figure 2. (Related to Figure 1)**

**A.** Box plots of the relative *SLC27A5* mRNA expression in normal liver and HCC tissues with different clinical stages (I, II and III) from two analyses in ONCOMINE database (*Jia Liver*, Clin Cancer Res, 2007; and *Wurmbach Liver*, Hepatology, 2007).

**B-C.** *SLC27A5* mRNA expression in *Wurmbach Liver* analysis was grouped by the presence of satellites (No and Yes) (B), or vascular invasion (microscopic and macroscopic) (C), and differences between groups were compared and shown by box plots. All values represented as mean ± SD, **P* < 0.05, ***P* < 0.01, and ****P* < 0.001 (two-tailed *t*-test).

**D.** Relative expression of FATP5 in 128 human HCCs with or without multiple tumors, high α-fetoprotein (> 200 ng/mL), and large tumor diameter (> 8cm). All values were expressed as mean ± SD, and n.s. represented as negative significance (*P* ≥ 0.05, two-tailed *t*-test).

**Supplementary Figure 3. (Related to Figure 2)**

**A.** Both the mRNA and protein expressions of FATP5 (*SLC27A5*) in 5 human HCC cell lines (SNU449, PLC/PRF5, MHCC97H, HCCLM3 and Huh7) and a hepatoblastoma cell line (HepG2) were evaluated by qRT-PCR and western blotting. The mRNA expression level was normalized to GAPDH (ΔCt) and compared with that in SNU449 cells (ΔΔCt; set as 1). The fold change was calculated and presented as 2^-ΔΔCt^.

**B-C.** qRT-PCR and western blotting of exogenous FATP5 expression in HCC cells transfected with empty Vector or FATP5 (MHCC97H and SNU449 cells), or infected with LV-shNC or LV-shFATP5 (sequence #1 or #2; Huh7, PLC/PRF/5 and MHCC97H-FATP5 cells). All mRNA values were expressed as the fold change relative to their corresponding control cells, with the expression levels presented as equal to 1. **P* < 0.05, ***P* < 0.01, and ****P* < 0.001 (two-tailed *t*-test). For all western blotting experiments in A, B and C, β-actin was used as internal loading control.

**D.** HEK-293T cells were transfected with HA-tagged FATP5 and FLAG-tagged AMPK (or Myc-tagged mTOR) expression constructs and cell lysates were prepared 48 hours later. Interaction of exogenous FATP5-HA with AMPK-FLAG (or mTOR-Myc) was determined by co-IP and western blot analyses with anti-HA and anti-FLAG (or anti-Myc) antibodies.

**Supplementary Figure 4. (Related to Figure 4)**

**A.** Both oxygen consumption rates (OCR) and extracellular acidification rates (ECAR) in SNU449 HCC cells were measured using a Seahorse XF96 Extracellular Flux Analyzer either under basal conditions or after treatment with according inhibitors (OLI: oligomycin; FCCP: carbonyl cyanide p-trifluoromethoxy- phenylhydrazone; Rtn/AA: rotenone/antimycin; 2-DG: 2-deoxyglucose). Representative curves were shown, and each data presented as mean ± SD, with the average value of 6-independent wells.

**B.** Basal and maximal respiration capacities (OCR rates) in MHCC97H and Huh7 HCC cells were presented as mean ± SD, ***P* < 0.01, and ****P* < 0.001compared with MHCC97H-Vec or Huh7-shNC cells, respectively.

**Supplementary Figure 5. (Related to Figure 5)**

**A-B.** Migration and Matrigel invasion assays were performed to evaluate the migratory and invasive capacities of SNU449-Vec or -FATP5 cells treated with or without Compound C or MHY1485 for 48 hours.

**C.** The lentiviral vectors expressing shRNAs for a non-targeted control (Santa Cruz, CA; sc-108080) or AMPKα1 shRNA (Santa Cruz, CA; sc-29673-V) were added to MHCC97H cell for 48 hours, and different stable clones expressing AMPKα1-specific shRNAs (clone #1 and #2) were selected by puromycin (1 μg/ml). The knockdown efficiency was determined by western blotting. β-actin was used as a loading control.

**D.** MHCC97H cells stably expressing Vec or FATP5 were transfected with shNC or shAMPKα1 (#2) for 48 hours. The migrated or invaded cells were microscopically counted and results were presented as the average number of cells from 5 independent microscopic fields. Scale bar: 50 μm. All data in A-B and D represented as mean ± SD, ***P* < 0.01.

**Supplementary Figure 6. (Related to Figure 5)**

Stable FATP5-overexpressing (SNU449 and MHCC97H) or FATP5 knockdown (MHCC97H-FATP5 cells expressing shNC and shFATP5 #1, #2) cells were stained with Annexin V and propidium iodide (PI) and the apoptosis rates were detected by flow cytometry.

**Supplementary Figure 1. (Related to Figure 1)**

**
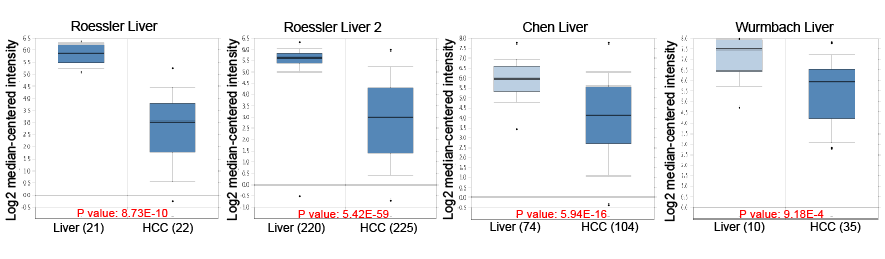
**

**Supplementary Figure 2. (Related to Figure 1)**

**
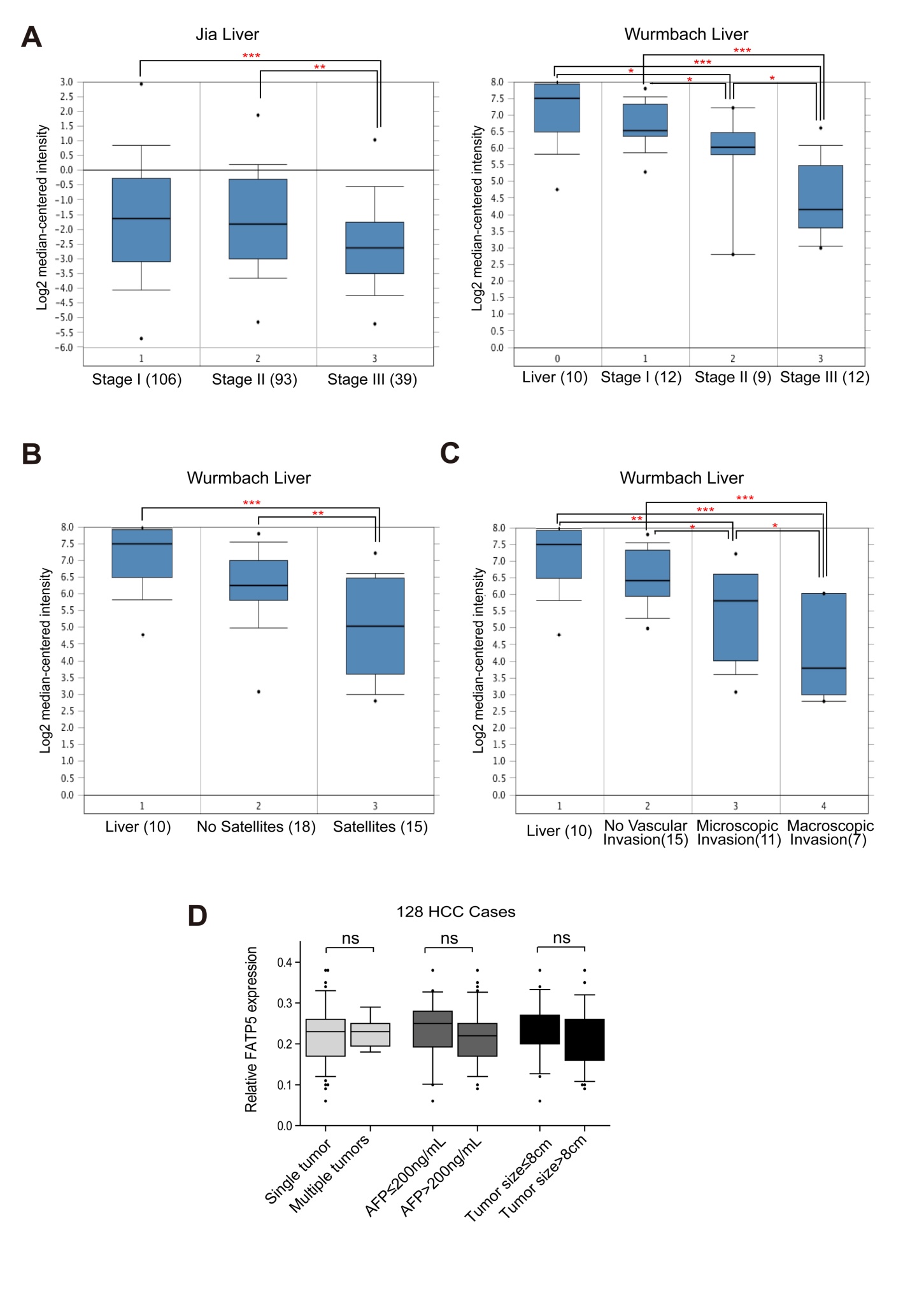
**

**Supplementary Figure 3. (Related to Figure 2)**

**
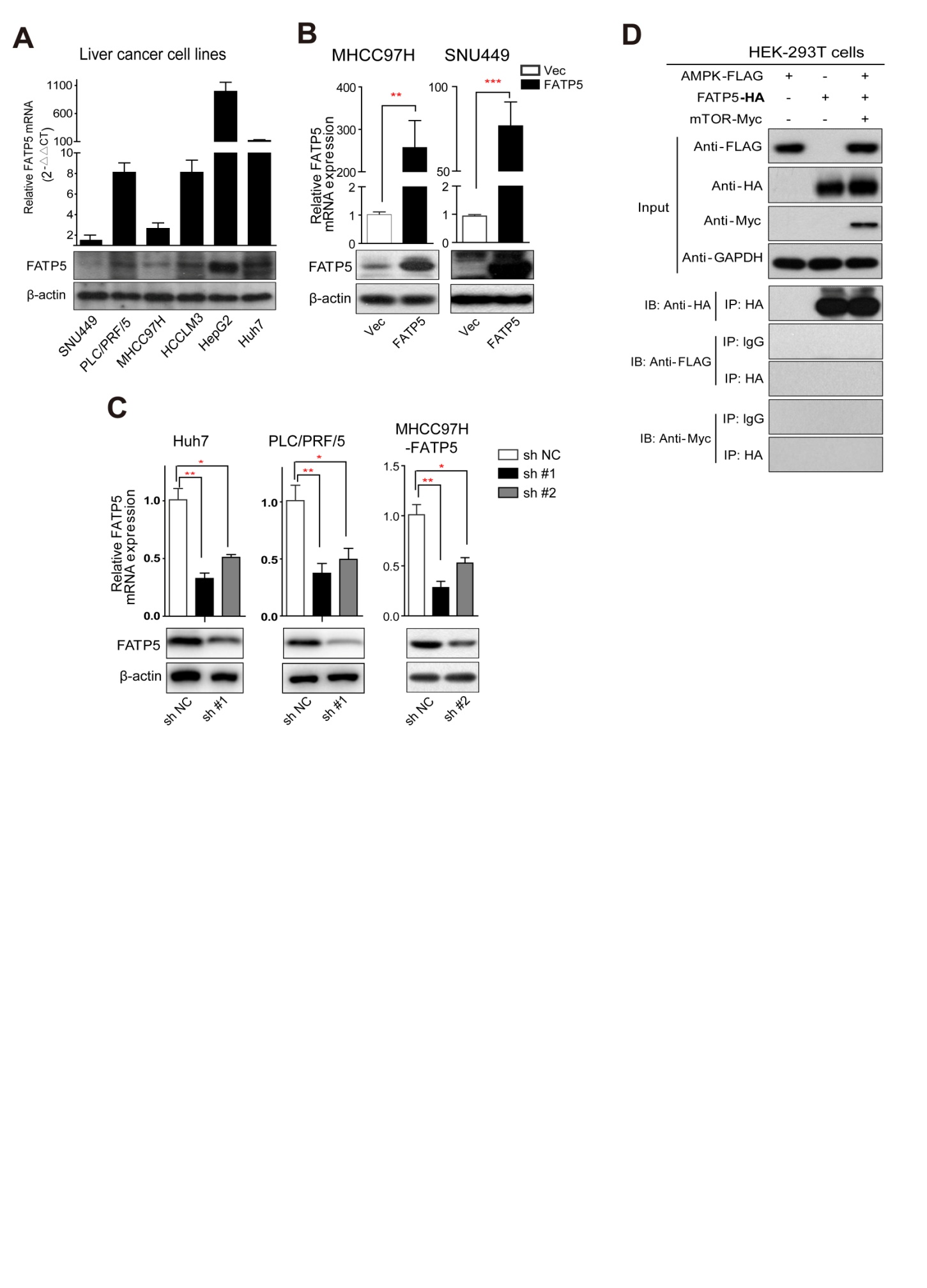
**

**Supplementary Figure 4. (Related to Figure 4)**


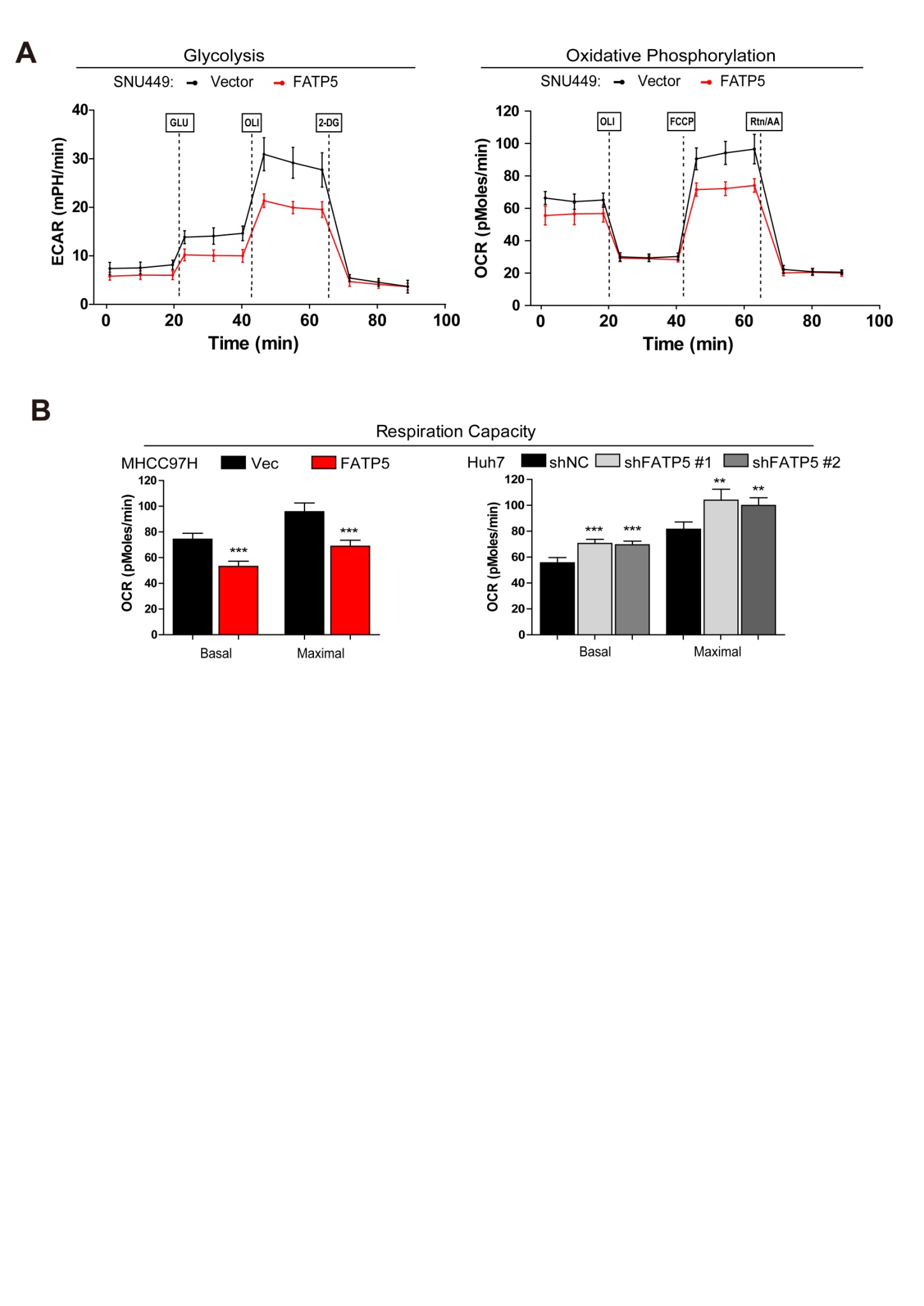


**Supplementary Figure 5. (Related to Figure 5)**


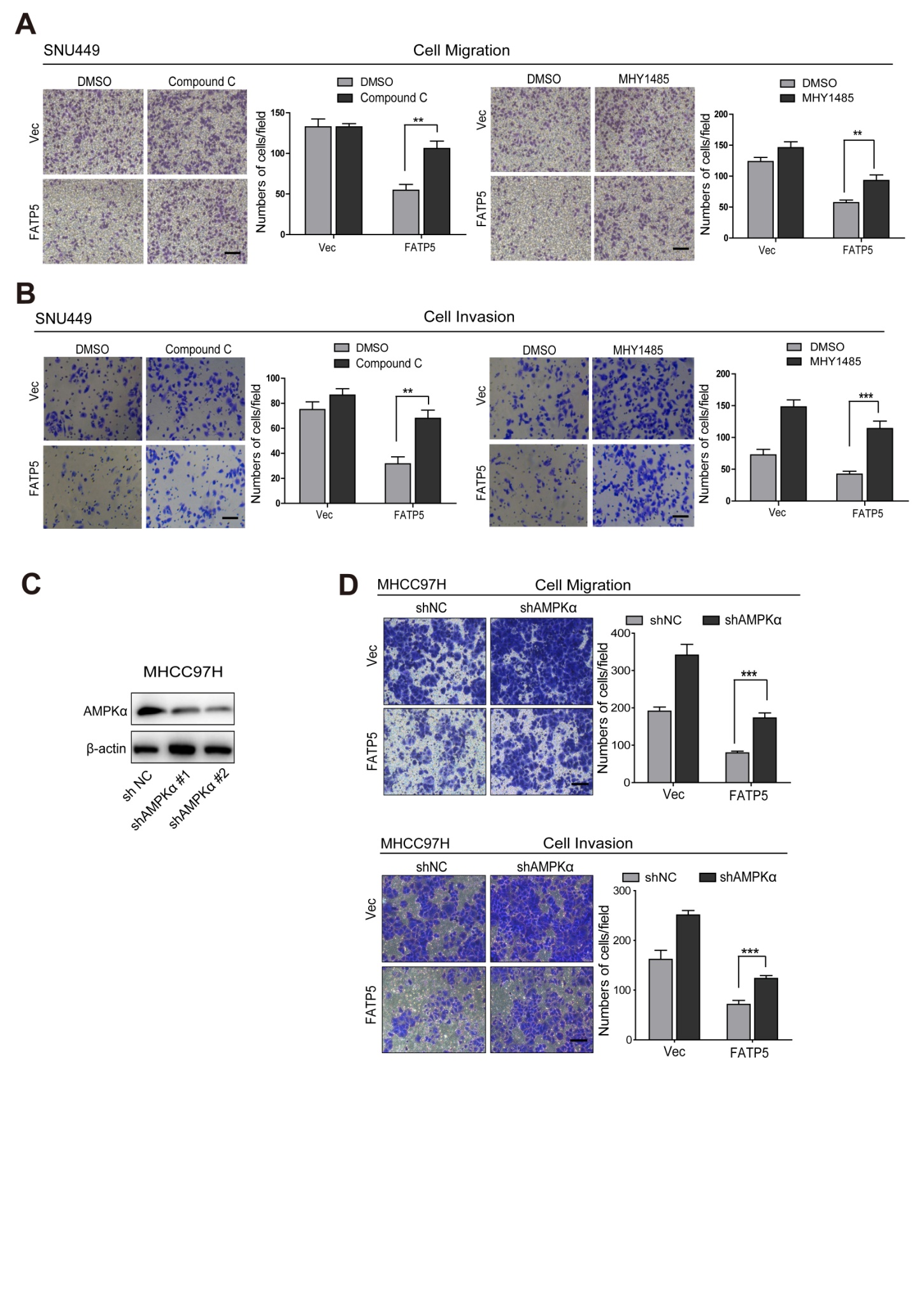


**Supplementary Figure 6. (Related to Figure 5)**

**
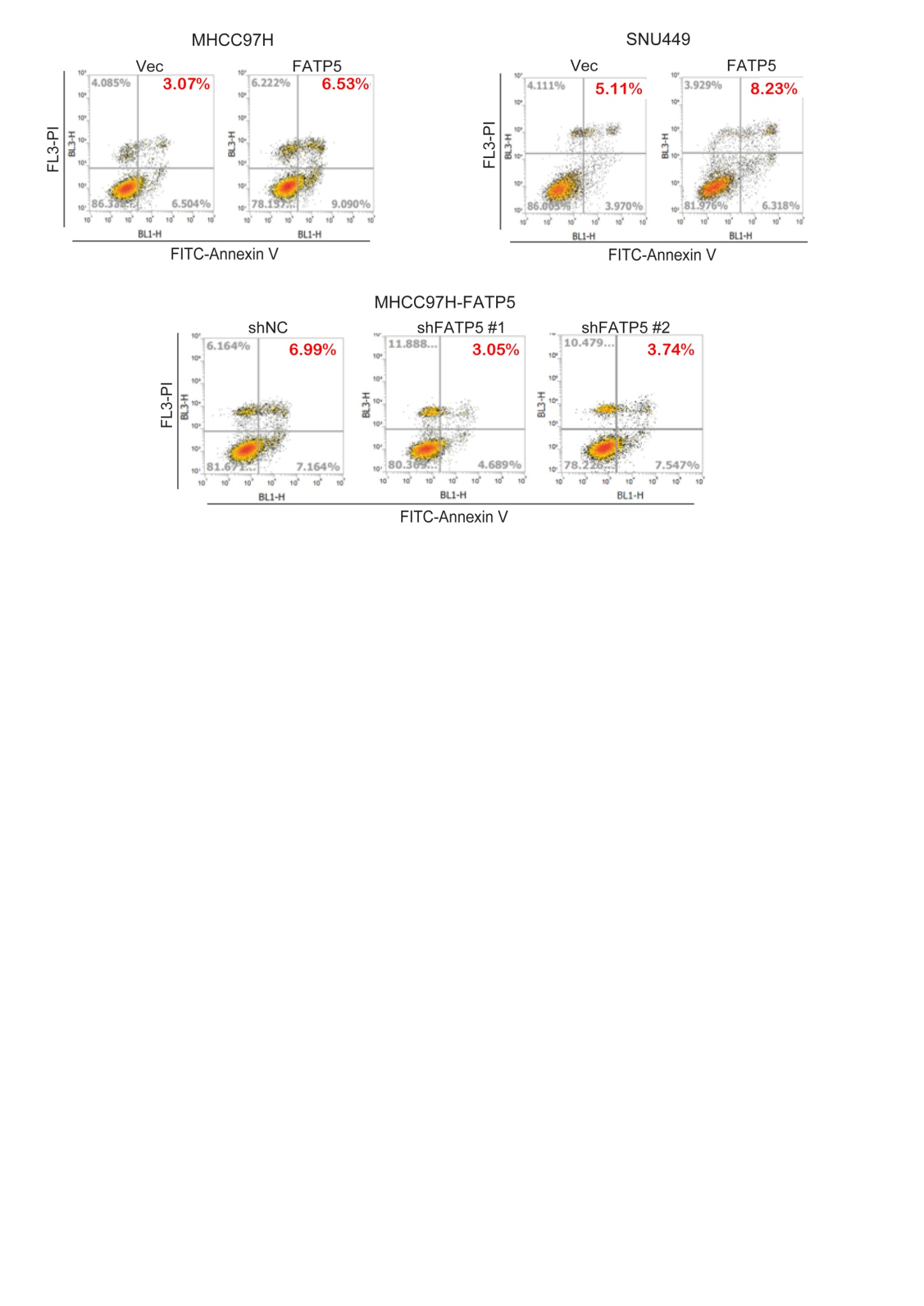
**

**Supplementary Tables**

**Supplementary Table 1.** Summary of the Clinicopathological Characteristics of 128 HCC Patients.

**Supplementary Table 2.** Correlation of Clinicopathological Characteristics with FATP5 Expression Levels.

**Supplementary Table 3.** Univariate and Multivariate Analyses of Prognostic Factors Associated with Recurrence and Survival.

**Supplementary Table 4.** Overview of HCC Databases.

**Supplementary Table 5.** Sequences for RNA Interference.

**Supplementary Table 6.** Primer Sequences for Real-time PCR.

**Supplementary Table 7.** Specific Primary Antibodies for Western Blotting.

**Supplementary Table 1. Summary of the Clinicopathological Characteristics of 128 HCC Patients.**

| Characteristics |  | Number of patients |
| --- | --- | --- |
|  |  |  |
| Patients |  | 128 |
|  |  |  |
| Age | ≤50 | 76 |
|  | >50 | 52 |
| Gender | Male | 104 |
|  | Female | 24 |
| Serum AFP (ng/mL) | ≤200 | 42 |
|  | >200 | 86 |
| Maximal tumor size (cm) | ≤8 | 74 |
|  | >8 | 54 |
| Tumor differentiation status | Ⅰ-Ⅱ | 29 |
|  | Ⅲ-Ⅳ | 99 |
| Tumor TNM stage | Ⅰ-Ⅱ | 58 |
|  | Ⅲ-Ⅳ | 70 |
| Tumor nodules number | Single | 119 |
|  | Multiple | 9 |
| Pathological microsatellites | Yes | 62 |
|  | No | 66 |
| Microvascular invasion | Yes | 90 |
|  | No | 38 |
| Macrovascular invasion | Yes | 61 |
|  | No | 67 |
| Tumor encapsulation | Complete | 32 |
|  | None | 96 |
| HBV association (HBs Antigen) | Positive | 107 |
|  | Negative | 21 |
| HBV association (HBe Antigen) | Positive | 17 |
|  | Negative | 111 |
| Liver cirrhosis | Yes | 91 |
|  | No | 37 |
| AFP, α-fetoprotein; HBV, Hepatitis B virus; TNM, Tumor Node Metastasis | | |

**Supplementary Table 2. Correlation of the Clinicopathological Characteristics with FATP5 Expression Levels.**

**
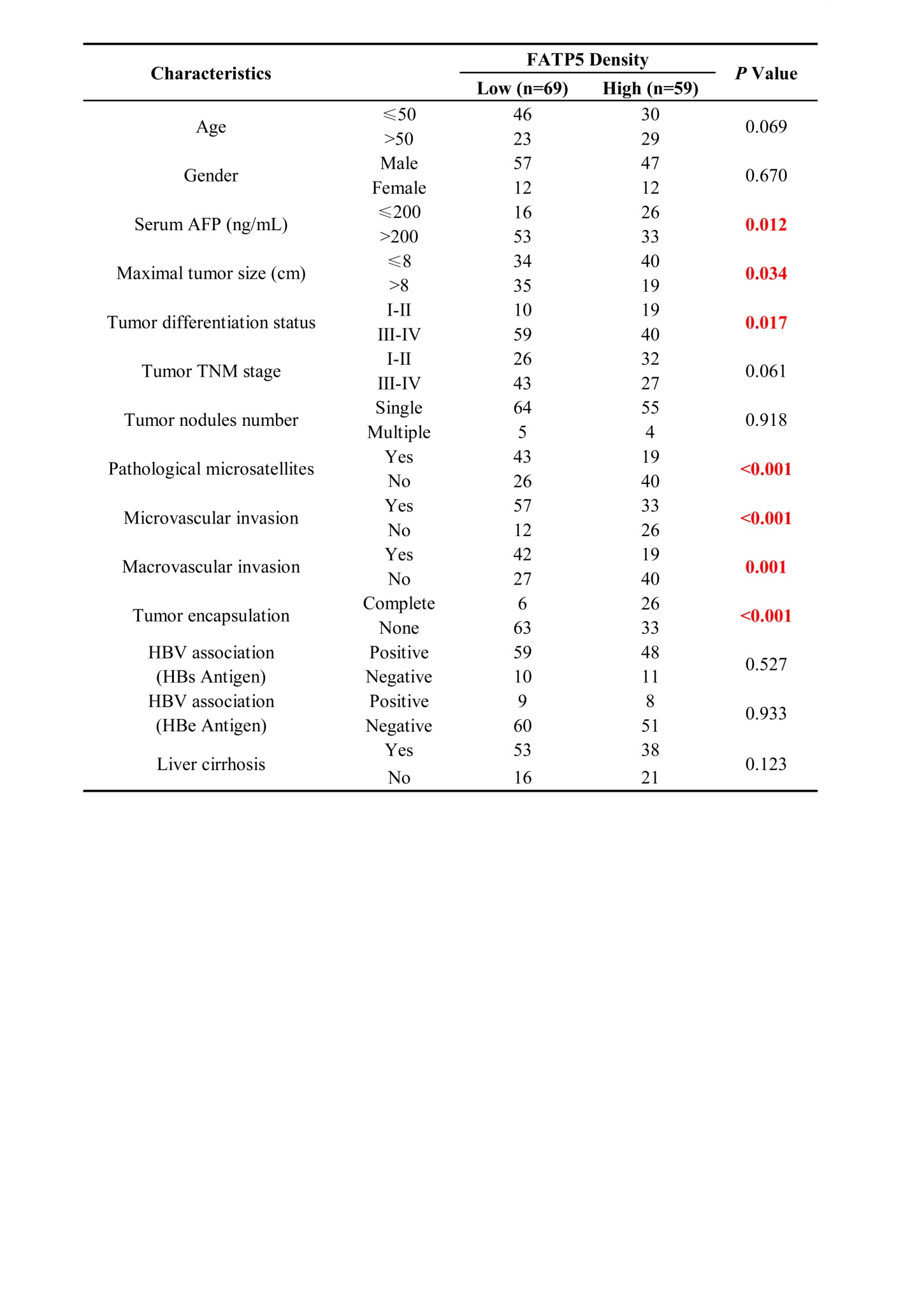
**

**Supplementary Table 3. Univariate and Multivariate Analyses of Prognostic Factors Associated with Recurrence and Survival.**

|  | |  | |  |  | |  |  |  | |  | | |  | |
| --- | --- | --- | --- | --- | --- | --- | --- | --- | --- | --- | --- | --- | --- | --- | --- |
| Variables | DFS | | | | | | | OS | | | | | | |  |
|  | Univariate | | Multivariate | | | | | Univariate | | Multivariate | | | | |  |
|  | *P* value | | *P* value | | HR | 95%CI | | *P* value | | *P* value | | HR | 95%CI | |  |
| TMAs assay (n=128) | | | | | | | | | | | | | | | |
|  |  |  |  |  |  |  |  |  |  |  |  |  |  |  |  |
| Gender (Female vs. Male) | | 0.294 | | NS |  | |  | 0.937 | NS | |  | | |  | |
| Age (≤50 vs. >50) | | 0.652 | | NS |  | |  | 0.061 | NS | |  | | |  | |
| AFP level, ng/ml  (≤200 vs. >200) | | 0.493 | | NS |  | |  | 0.273 | NS | |  | | |  | |
| Maximal tumor size, cm  (≤8 vs. >8) | | <0.001 | | NS |  | |  | <0.001 | NS | |  | | |  | |
| Tumor nodules number  (Single vs. Multiple) | | 0.675 | | NS |  | |  | 0.897 | NS | |  | | |  | |
| Microvascular invasion  (No vs.Yes) | | <0.001 | | **0.003** | 2.514 | | 1.369-4.618 | <0.001 | **0.045** | | 1.966 | | | 1.014-3.813 | |
| Macrovascular invasion  (No vs.Yes) | | <0.001 | | **0.015** | 1.837 | | 1.128-2.994 | <0.001 | **0.035** | | 1.771 | | | 1.040-3.013 | |
| Encapsulation  (Complete vs. None) | | 0.032 | | NS |  | |  | 0.059 | NS | |  | | |  | |
| Pathological microsatellites (No vs. Yes) | | <0.001 | | **0.005** | 1.932 | | 1.224-3.048 | <0.001 | **0.005** | | 1.982 | | | 1.223-3.212 | |
| Tumor differentiation  (I-II vs. III-IV) | | <0.001 | | NS |  | |  | 0.001 | **0.036** | | 1.919 | | | 1.045-3.523 | |
| Tumor TNM stage  (I-II vs. III-IV) | | <0.001 | | NS |  | |  | <0.001 | NS | |  | | |  | |
| Liver cirrhosis  ( No vs. Yes) | | 0.611 | | NS |  | |  | 0.606 | NS | |  | | |  | |
| HBs antigen  (Negative vs. Positive) | | 0.622 | | NS |  | |  | 0.723 | NS | |  | | |  | |
| Hbe antigen  (Negative vs. Positive) | | 0.555 | | NS |  | |  | 0.311 | NS | |  | | |  | |
| FATP5 (High vs. Low) | | <0.001 | | **0.029** | 1.655 | | 1.053-2.603 | <0.001 | **0.021** | | 1.759 | | | 1.087-2.845 | |

Univariate analyses were calculated by the Kaplan-Meier method (P<0.05 by log-rank test). Those variables found significant at P < 0.1 in univariate analyses were entered into multivariable Cox-regression analyses.

FATP, Fatty acid transport protein; TMA, Tissue microarray; TNM, Tumor Node Metastasis; OS, Overall survival; DFS, Disease-free survival AFP, α-fetoprotein; HR, Hazard ratio; CI, Confidence interval; NS, Not significant.

| **Supplementary Table 4. Overview of the HCC Datasets** | | | |
| --- | --- | --- | --- |
| datasets | Samples | | Platform |
|  | Non-Tumor | Tumor |  |
| GSE22058 | 97 | 100 | mRNA array |
| GSE54236 | 80 | 81 | mRNA array |
| GSE36376 | 193 | 240 | mRNA array |
| TCGA | 50 | 349 | RNA-seq |

**Supplementary Table 5. Sequences for RNA Interference**

| No. | Sense Sequence (5' to 3') |
| --- | --- |
| shRNA NC | CCTAAGGTTAAGTCGCCCT |
| FATP5 shRNA #1 | GCGTGACAGTGATCCTGTA |
| FATP5 shRNA #2 | CCTGTAGCAGTGGCAGAAT |
| FATP5 shRNA #3 | ACAGCTGATGATGTGGTTT |
| FATP5 shRNA #4 | AGCACGTTCAAACTGATGA |

**Supplementary Table 6. Primer Sequences for Real-time PCR.**

| Primer | Sequence (5' to 3') |
| --- | --- |
| h-GAPDH-forward | GGAGCGAGATCCCTCCAAAAT |
| h-GAPDH-reverse | GGCTGTTGTCATACTTCTCATGG |
| h-FATP5-forward | CATGGCGTGACAGTGATCCT |
| h-FATP5-reverse | CAGCCCGTAGTCCATTGCC |
| h-E-cadherin-forward | GACGCGGACGATGATGTGAAC |
| h-E-cadherin-reverse | TTGTACGTGGTGGGATTGAAGA |
| h-N-cadherin-forward | GCTTATCCTTGTGCTGATGTTT |
| h-N-cadherin-reverse | GTCTTCTTCTCCTCCACCTTCT |
| h-Vimentin-forward | CCGACACTCCTACAAGATTTAGA |
| h-Vimentin-reverse | CAAAGATTTATTGAAGGAGAACC |

**Supplementary Table 7. Specific primary antibodies for Western Blotting.**

| Antibody | Dilution | Application | No. | Company |
| --- | --- | --- | --- | --- |
| FATP5 | 1:1000 | WB | ab224069 | Abcam, Cambridge, UK |
| FATP5 | 1:200 | IHC | ab224069 | Abcam, Cambridge, UK |
| Ki-67 | 1:400 | IHC | 9027 | Cell Signaling, MA, USA |
| total AMPKα | 1:1000 | WB | 2532s | Cell Signaling, MA, USA |
| p-AMPKα (Thr172) | 1:1000 | WB | ab133448 | Abcam, Cambridge, UK |
| Total mTOR | 1:1000 | WB | 2983T | Cell Signaling, MA, USA |
| p-mTOR (Ser2448) | 1:1000 | WB | 5536T | Cell Signaling, MA, USA |
| total ACCα | 1:1000 | WB | 21923-1-AP | Proteintech, IL, USA |
| p-ACCα (Ser79) | 1:1000 | WB | 11818s | Cell Signaling, MA, USA |
| total p70 S6K | 1:1000 | WB | 14485-1-AP | Proteintech, IL, USA |
| p-p70 S6K (Thr389) | 1:1000 | WB | 9234 | Cell Signaling, MA, USA |
| S6 | 1:1000 | WB | 14823-1-AP | Proteintech, IL, USA |
| p-S6 (Ser235/236) | 1:1000 | WB | 2211 | Cell Signaling, MA, USA |
| E-cadherin | 1:1000 | WB/IHC | 3195T | Cell Signaling, MA, USA |
| N-cadherin | 1:1000 | WB/IHC | 13116T | Cell Signaling, MA, USA |
| Vimentin | 1:1000 | WB | 5741T | Cell Signaling, MA, USA |
| HA-tag | 1:50 | IP | ab236632 | Abcam, Cambridge, UK |
| FLAG-tag | 1:50 | IP | ab125243 | Abcam, Cambridge, UK |
| Myc-tag | 1:50 | IP | ab32 | Abcam, Cambridge, UK |
| GAPDH | 1:20000 | WB | 5174T | Cell Signaling, MA, USA |
| β-actin | 1:5000 | WB | Ab8227 | Abcam, Cambridge, UK |
